# Complete response to intravesical gemcitabine in non‐muscle invasive bladder cancer patient after BCG failure: A case report and literature review

**DOI:** 10.1002/ccr3.6373

**Published:** 2022-09-22

**Authors:** Fouad Nahhat, Modar Doyya, Hazem Ksiri

**Affiliations:** ^1^ Faculty of Medicine Damascus University Damascus Syria; ^2^ Department of Oncology Albairouni University Hospital Damascus Syria

**Keywords:** BCG failure, bladder cancer, case report, gemcitabine, intravesical, NMIBC

## Abstract

Bladder cancer treatment remains a challenge to every oncologist. We report the case of a 57‐year‐old man with BCG‐refractory bladder cancer who had a complete response to intravesical gemcitabine to highlight the role of gemcitabine as a bladder sparing treatment in BCG‐failure patients.

## 1 INTRODUCTION

Bladder cancer is the 10th most common cancer worldwide and has a steadily increasing incidence.^1^ It is more common among men, for whom it ranks as the 6th most diagnosed and the 9th most fatal cancer.^1^ At presentation, 75% of cases are confined to the urothelium or lamina propria (nonmuscle invasive bladder cancer, NMIBC).^2^ Then, the initial treatment includes a transurethral resection (TUR), followed by an intravesical adjuvant therapy with Bacillus Calmette–Guérin (BCG) for intermediate and high‐risk patients.^3^ In case of BCG failure, radical cystectomy is the standard of care in high‐risk patients. However, many of them are unfit or they refuse to undergo such an intervention; therefore, other treatment options are required.^4^ Herein, we report the case of a bladder cancer patient who had a complete response to intravesical gemcitabine after BCG failure, to highlight the potential effectiveness of gemcitabine as a bladder sparing treatment in BCG‐failure patients who cannot undergo or refuse surgery.

## 2 CASE PRESENTATION

A 57‐year‐old man, 45 pack‐year smoker, presented to the clinic complaining of gross hematuria. His medical history was significant for diabetes mellitus, peripheral vascular disease, cardiomyopathy, and prior cerebral hemorrhage. Also, he had underwent a coronary artery bypass graft (CABG) in September 2019. Otherwise, his clinical examination was insignificant.

**FIGURE 1:**
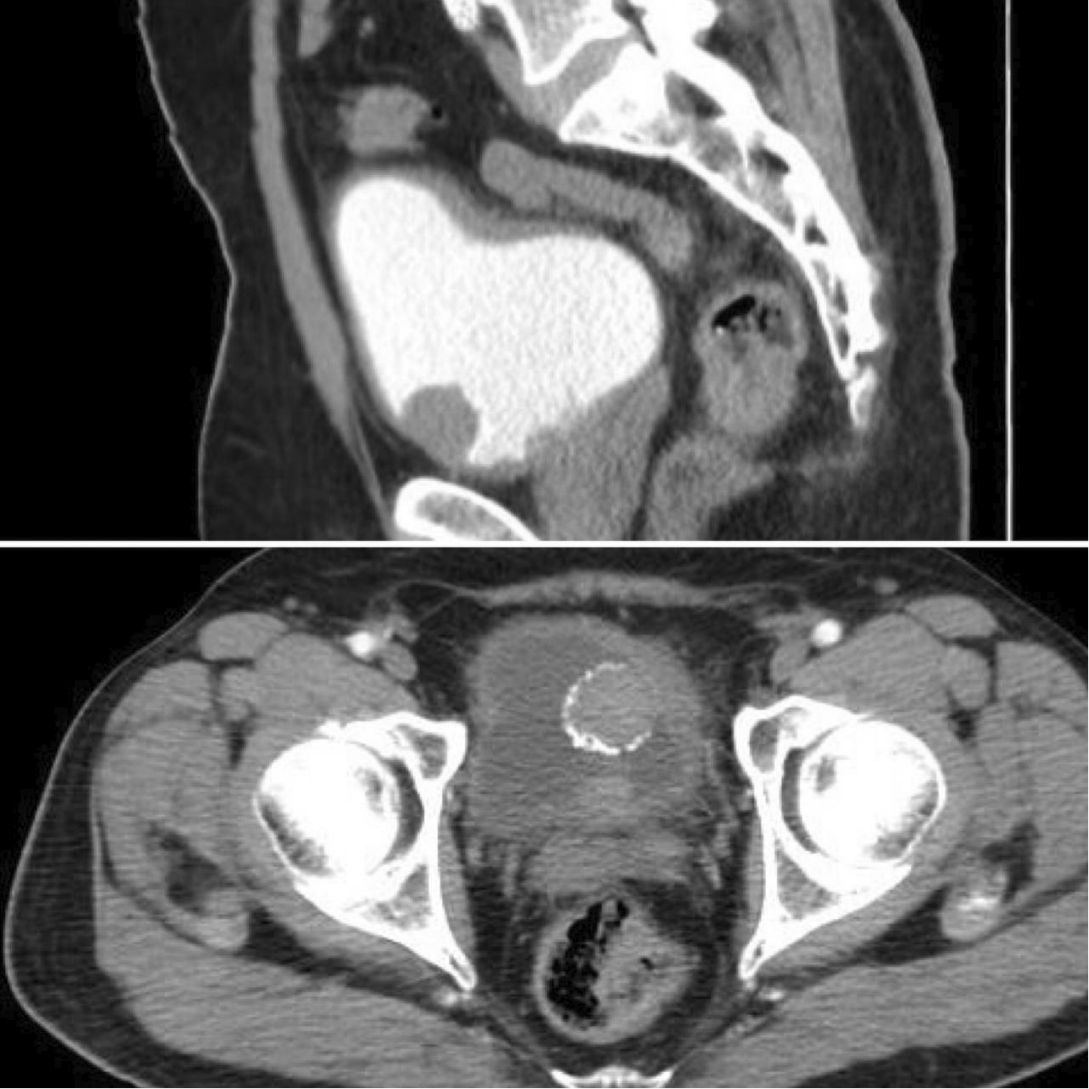
The CT scan shows the multifocal bladder tumor

The cystoscopy and CT scan (shown in Figure 1) showed a multifocal bladder tumor. Transurethral resection (TUR) was performed. The TUR pathology revealed a stage‐T1G3 transitional cell carcinoma, and the muscularis was present but free of tumor; therefore, non‐muscle invasive bladder cancer (NMIBC) was diagnosed. The tumor involved the prostatic urethra. The CT scan excluded any distant metastases.

After that, the patient received one vial of intravesical Bacillus Calmette‐Guerin (BCG)‐Medac™ once a week for 6 weeks. Three months later, the TUR showed residual high‐grade T1 tumor fragments; therefore, the patient was considered BCG‐refractory.

Radical cystectomy is the standard of care in such situations. However, due to the cardiomyopathy, the patient was unfit for the surgery and also refused it. So, other treatment options were required.

Another TUR was performed and followed by the intravesical injection of 2 g of Gemcitabine once a week for 6 weeks. After 3 months, the cystoscopy and taken biopsies showed complete response to the treatment and no evidence of tumor. No side effects were encountered during the therapeutic course.

## 3 DISCUSSION & CONCLUSION

Non‐muscle invasive bladder cancer (NMIBC) remains a therapeutic challenge, especially in the era of BCG shortage. Although the transurethral resection (TUR) of the tumor followed by intravesical BCG injection has long been the standard of care for NMIBC, the treatment fails in about 40%–50% of patients.^5^

The classifications of BCG failure are shown in Table 1.^6^, ^7^

**TABLE 1:** BCG failure classifications

References	Classification	Description
Kamat 2016^6^	BCG refractory	Persistent high‐grade disease at 6 months after adequate BCG treatment or any stage or grade progression by 3 months after the first BCG cycle. For example, high‐grade disease recurrent at 3 months after initial Ta, T1, high‐grade disease, or CIS).
	BCG relapsing	Recurrence of high‐grade disease after achieving a disease‐free state for 6 months after adequate BCG induction and maintenance therapy.
	BCG intolerant	Disease persistence due to patient's inability to receive adequate BCG treatment.
Martini 2017^7^	BCG unresponsive	BCG refractory or relapsing as mentioned above occurring within 6 months of last BCG exposure for patients receiving maintenance therapy. This group of patients are at highest risk of recurrence and progression.

Radical cystectomy is indicated in cases of BCG failure and provides a 92% disease‐free survival when performed early.^8^ However, post‐surgical quality of life assessment showed many physical, mental and social health problems in patients who underwent the surgery.^9^ So many people refuse such intervention. On the other hand, many of them are unfit for surgery due to cardiac or other health issues.

As an alternative to surgery, bladder‐sparing treatments include a second course of BCG, intravesical mitomycin C (MMC), intravesical chemotherapy with gemcitabine and a few other options.^10^

Gemcitabine (GEM) has now level‐one evidence as an effective drug for bladder cancer.^11^ When used intravesically, GEM reaches low plasma levels, which reduces systemic toxicity.^12^

A systematic review and meta‐analysis compared the efficacy and safety of intravesical GEM versus MMC for NMIBC and demonstrated that using GEM is associated with a statistically significant decrease in tumor recurrence rate and reduction in local toxicity compared with MMC.^13^ In addition, MMC is an expensive drug that cannot be affordable in some low‐income countries.

Ye et al.^14^ conducted a similar meta‐analysis on five clinical trials with an overall 386 bladder cancer patients, comparing GEM to BCG. The results showed no statistically significant difference in tumor recurrence rates, but GEM was associated with significantly lower rates of dysuria and hematuria in comparison with BCG.

Our patient suffers from severe cardiomyopathy that makes surgery contraindicated. He also refused the radical cystectomy due to the poor postoperative quality of life.

Considering the reasons mentioned above, we preferred GEM over other treatment options after the first BCG failure. The treatment course led to a complete pathologic response with no side effects. A 6‐month follow‐up showed no tumor recurrence, but a longer follow‐up time is needed to determine the long‐term efficacy of the treatment.

In our case, we aim to highlight the promising role of GEM in treating resistant bladder cancers and avoiding radical cystectomy complications.

Since our study was performed on one patient only, it provides relatively weak—but important—evidence. So, to formulate definitive recommendations, larger and higher‐quality studies are required.

## AUTHOR CONTRIBUTIONS

Fouad Nahhat: wrote the abstract, introduction, and discussion and participated in the literature review. Modar Doyya: wrote the case presentation, designed the figure, and participated in the literature review. Hazem Ksiri: participated in the patient's treatment and supervised the manuscript preparation, scientifically and academically.

## FUNDING INFORMATION

There were no funding sources.

## CONFLICT OF INTEREST

The authors declare no conflict of interest.

## ETHICAL APPROVAL

Ethical approval is not required for this study in accordance with local or national guidelines.

## CONSENT

Written informed consent was obtained from the patient to publish this report in accordance with the journal's patient consent policy.

## Data Availability

All data are included in this article. Further enquiries can be directed to the corresponding author.
